# Editorials

**Published:** 1890-02

**Authors:** 


### HOMŒOPATHIC PHYSICIAN,

A MONTHLY JOURNAL OF

HOMŒOPATHIC MATERIA MEDICA AND CLINICAL MEDICINE.

If our school ever give up the strict inductive method of Hahnemann, we are lost, and deserve only to be mentioned as a caricature in the history of medicine."—CONSTANTINE HERING.

Vol. X. FEBRUARY, 1890. No. 2.

### EDITORIALS.

LA GRIPPE.—As the literature of "la grippe" comes along in the journals, we poor benighted followers of Hahnemann may learn what heaps of science *and* drugs are required for its treatment.

While we are keeping our deluded patients from the undertakers' hands; and while we are not helping to fill the coffers of drug venders, our scientific friends are still theorizing about the pathological character of the disease, and are as much at variance regarding its treatment as they always are in the treatment of any affection.

Thus, Dr. Dobell, of London, advises: "The bowels to be kept open by any aperient known to act most kindly with the patient." Then the following inhalation: "Creasoti, ʒj ; ole' caryoph., ʒj ; olei eucalyp. glob., ℥j. ; tr. camph. co. ad., ℥ij.'i "To be put in a jar with boiling water and inhaled, and repeated every two hours. In the interim gargle occasionally with hot water. Soon after the first inhalation begin the following restorative febrifuge: sp. camphoræ, ℥j. ; sp. etheris nitr., ℥j. ; tr. quin. ammon. ad ℥ij. Ft. Guttæ. One teaspoonful stirred in a claret-glass of water every three hours. If the temperature rises above 101° F., take gr. v. of antifebrin in two tablespoonfuls of beef-tea, and repeat every hour until the temperature falls to 100°, continuing the inhalations and drops as before. If pain or oppression at the chest sets in, apply hot poultices." That is the *English of it.*

Our French friends, speaking through M. Henri Huchars, in the *Revue généralé de Clinique et de Thérapeutique*, think that adynamic symptoms are the most marked. Cinchona, alcoholic beverages, and the like are recommended to be given at first. "For more serious cases, where prostration is marked, injections of ether or of caffeine should be used. If the fever continues after quinine, eight to fifteen grains, has been given, then fifteen grains of antipyrin should be taken. If rheumatoid and neuralgic symptoms, the antipyrin should be given two or three times a day. Or, in the place of antipyrin, phenacetine aud salol, in eight-grain doses. When the adynamia becomes more marked, from broncho-pulmonary complications, stimulants are to be used freely. Strychnia is advised when dyspnœa is extreme. And, in the renal form of the affection, sometimies, the only thing that will relieve is venesection, though, ordinarily, this is the last thing to be thought of in a disease in which the adynamic tendency is so pronounced. In the gastro-intestinal form it is necessary to employ mild purgatives, such as castor-oil or calomel, and to obtain intestinal antisepsis by means of salicylate of bismuth, or of magnesia, naphthol, etc. If an emetic is at any time indicated, tartar-emetic should never be used, but ipecac given in preference."

If anything, we think the French slightly ahead of the English. Please note the Englishman says nothing of "intestinal antisepsis," and nothing of venesection. We are standing up for both of these procedures. How can any one recover whose intestines need "antisepsis" and do not get it?

And then the stimulants! Who will not stand up for stimulants? It is true that they do render the chance of recovery less; that they are a draught on vitality; and that the patient is usually made more adynamic by their use. What of that? Are you not doing something for the patient? And when death comes can you not have all who knew of the treatment of the case bear witness that you have done all that is possible? Mongrels, make note of this. The two schools are coming closer together, and even though you do take the name of beneficent Homœopathy, do not hesitate to do as you always do—what is possible.

After doing what is possible, and when your patients are buried—for you are the cause of burying many who think you practice Homœopathy—then, if you have any conscience, and wish to know how to do right, go to the works of Hahnemann, and learn of him how to apply the only law of therapeutics. Then you can do only what is right; then you will more plainly see what harm you are doing to a principle which has its foundation in honesty, and a superstructure of results that stand unapproached in the history of medicine. G. H. C.

---

WINES AND LIQUORS IN MEDICINE.—Alcohol, in its various forms, as brandy, wines, etc., can be used in cases in which it is indicated according to the law of similars, with as good results as other remedies show.

Brandy-drinkers are liable to cancer of the stomach, in which there is intense burning pain. A few drops of brandy in half a tumblerful of water will relieve, temporarily, this intense burning. Drinkers of Rhine wines are prone to bladder troubles. Of this fact we may make use. Madeira wines cause affections of the heart. A spoonful of Madeira wine will often relieve the more grave symptoms of organic heart disease.

The continued use of whiskey, as is well known, causes cirrhosis of the liver and of the kidneys, and gives rise to a train of symptoms which should be sufficient to keep any one with this knowledge from becoming a whiskey-drinker.

No one, with even a superficial knowledge of Homœopathy will ever use stimulants in any form in disease, as only harm can result. But where poverty of the blood exists there is nothing to compare to red wines, and our own country now furnishes as good wines as can be found. Egg Harbor, New Jersey, now produces wines equal to Burgundy in being full-bodied and generous, and we know of no native wines that are at all comparable with them. This subject deserves fuller treatment, and we trust that we may shortly be able to go into it more deeply.

G. H. C.

---

MERCURIUS IN TYPHOID FEVER.—In Hering's *Condensed Materia Medica*, under Chill, in Mercurius, is this symptom: Contra-indicated in typhoid fever, except for marked icteroid or scorbutic symptoms. In the *Guiding Symptoms* there is this: Typhoid fevers with marked icteroid or scorbutic symptoms. Among some of the older Hahnemannians the same idea obtained. Upon what it was based we were never able to learn. Some years ago, while treating a severe case of typhoid fever, we called the late Dr. Fellger in consultation. At the time the symptoms called for Mercurius, and we were giving it, although we knew of the above in Hering's *Condensed*. Dr. Fellger confirmed our choice of remedy. We then spoke to him of Hering's observation. He informed us that he knew of no reason for not giving Mercurius in typhoid fever, when indicated. Dr. Fellger said he had spoken to Dr. Hering on the subject, and that Hering thought it should not be given. Dr. Lippe seems to have had the same idea. Dr. Fellger related a case of typhoid fever, which he had seen many years before, in consultation with Dr. Lippe. Fellger said Mercurius was indicated. Lippe was averse to giving it. Dr. Fellger at last convinced him that as it was indicated nothing else should be given. The case was a desperate one, and after the Mercurius was given the serious condition began to improve, and the patient made a good recovery. It is the duty of the homœopathician always to give the indicated remedy, no matter what the condition. Otherwise we should be as hopelessly muddled as are the mongrels.

G. H. C.

---

ANTISEPTICS.—"Who shall ever know the number of victims that have been slain in the large cities, in hospital and private practice, on the altar of this Moloch of antisepsis?" This is a query found in a letter from "a country doctor" in the *Medical Record*, Dec. 28th, 1889. It is a pertinent question. In view of the practice now common of using so-called antiseptics in cases of wounds, operations, and even in obstetric practice for normal labor, it seems time to call a halt. If such be necessary in labor, which is a physiological process, why should we not resort to their use when we take food, and at all other times? If it be true that microbes cause all the affections we meet, why should we not constantly live in an atmosphere made sterile (?) by the use of antiseptics? If Lawson Tait's experience has not been sufficient to show the utter nonsense of this craze, we Hahnemannians can bring sufficient testimony to bear to convince any thinking person that not only is there no necessity for such treatment, but much harm is done, and death frequently follows the use of Carbolic acid and bichloride of Mercury, the substances most often used.

Lister, the father of antisepticism, not wishing to turn aside from the usual method of lauding highly, for a time, a plan for treating surgical affections, has concluded to drop what he has been recommending for years, and has taken to a new antiseptic, the double cyanides of Mercury and Zinc. The users of this will soon get up provings of this double salt, and we can then profit by their work.

In making use of a mixture of remedies we are still following Hahnemann's teachings. It does not matter how many substances enter into any compound, so long as we can have a record of the proving of that mixture, and so long as we can use preparations made from the original. S. L. asks a question on this subject at p. 353, Aug., 1889, number of this journal, and wishes to know "whether it shall be considered progressive Homœopathy or otherwise." It is certainly Homœopathy, and Homœopathy is always progressive. That which is a fact in Homœopathy to-day will be a fact for all time. Law to govern and guide in the practice of medicine, an unchangeable law of Nature, is what enables us to be progressive. Without law no advance is made. *Vide* the morass of allopathy and mongrelism.

G. H. C.

---

Microbes, Bacilli, Cocci, these be the cry of scientific medicine—at present. How long will they continue to afford the scientists ground for their ignorance? Give a moment's *homœopathic* thought to the subject, and bring your experience to bear. It is assumed that each disease has its microbe; and that the organisms can be cultivated; and that the same disease can be generated by inoculating this cultivation. Q. E. D. There is doubt regarding it even in the ranks of those who make the claim. Take tuberculosis, for illustration. How often have you seen tuberculosis where there was no history of heredity? Doubtless you may cause disease by inoculating the cultivations; but, are you not introducing some septic substance, which is the prime factor in causing the symptoms of disturbance? Where tuberculosis does occur with a want of heredity to account for the condition, do not stop there, but question, and you will learn that the mode of life has been such as to get the system in such a state as to cause the condition present. Microbes, and all other so-called generators of disease are vegetable parasites, and like all vegetable life, will only flourish on a suitable soil. Even hereditary tuberculosis may be made to remain latent by living properly, and living properly means, above all other things, plenty of wholesome air and nourishing food. Live as a civilized man should, and do not defile your lungs with pre-breathed air—the most prolific source of not only affections of the lungs, but of all other maladies. G. H. C.

---

Hahnemannian Homœopathy.—Nothing requires more firmness than to practice Hahnemannian Homœopathy properly. The choice of the remedy in a given case may be easy, but to watch its effects, and to permit it to do all that it properly can do, and at the same time to have the patient's friends continually asking, and advising the physician to do more, or something else, calls for an amount of resolution that many men lack. Nothing is more difficult than an attempt to show a patient or his friends that it is necessary to await the action of one remedy before resorting to another. Particularly in serious and painful affections is this the case. And yet, knowing that one is doing, not what is possible, but right, it is one's conscientious duty not to be turned from that path. The Hahnemannian, if he has chosen the remedy after a careful and painstaking study of all the elements of the case, and if he bear in mind the teachings of those who have given a lifetime to the study and practice of Hahnemannian Homœopathy, should know that there is literally no other way of doing good to his patient.

If we wish to know what Hahnemann taught on this subject, we may read these words: "If the medicine we have chosen for the positive (curative) treatment excites almost no sufferings previously unfelt by the patient, produces no new symptom, it is the appropriate medicament, and will certainly cure the original malady, even though the patient and his friends should not admit that any amendment has resulted from the commencing doses—and so, also, conversely, if the amelioration of the original disease take place in its whole extent from the action of the curative medicine, the medicine cannot have excited any serious new symptoms.

"Every aggravation, as it is called, of a disease that occurs during the use of a medicine (in doses repeated before or immediately after the expiry of its term of action), in the form of new symptoms not hitherto proper to the disease, is owing solely to the medicine employed (if it do not occur just a few hours before inevitable death, if there have taken place no important error of regimen, no outbreak of violent passions, no irresistible evolution of the course of nature by the occurrence or cessation of the menstrual function, by puberty, conception, or parturition); these symptoms are always the effect of the medicine, which, as an unsuitably chosen positive remedy, or as a negative (palliative) remedy, either ill selected or given for too long a time and in too large doses, develops them by its peculiar mode of action to the torment and destruction of the patient."

This from Hahnemann's *Lesser Writings*. This is a work that every practicing physician should possess. It contains much written subsequent to the *Organon*, and much is to be learned from it that can be found in no other place. From its pages we may readily see what a profound thinker we follow, and no homœopathician's education is complete without a knowledge of its contents. G. H. C.

Pathology.—If those so-called homœopathists who seem so anxious to don "pathological livery," as our lamented Lippe used to say, would only familiarize themselves with all that is said on the subject of pathology by those who most cultivate that field, possibly they might be made to see that symptomatology, in the Hahnemannian sense, is of more value. The President of the New York Pathological Society said, at a recent meeting: "As yet pathology has advanced medical science but little toward its true object, the cure of disease, but I believe that the time is coming when this object will be attained, and when, by a more perfect knowledge of the beginnings of disease, its treatment will be more rational and more satisfactory in its results."

Pathology, relegated to its proper place; used as it should be used; used as only a Hahnemannian knows how rightly to use it, is of assistance only in enabling the true physician to approximately estimate the progress of the disease, and the value of the action of the remedy.

Even though we were to admit that pathology can assist us in treating any given case, can we also admit that the most of what is now given as pathology is of any real, practical value? Pathology to be of worth should be the *natural history* of diseases. And what is known of diseased states, minus the alterations in the various tissues caused by the use of powerful drugs: and these mostly in conglomeration? Literally nothing. Even histology lacks much that it otherwise would possess if it had been elaborated by study of the tissues uncomplicated and unaltered by drugs. There is more to be expected from pathology, viewed solely as a knowledge of diseases, when we know that what is offered as such is a *real* natural history of disease. Until then, those who vaunt themselves as possessing such knowledge should be more modest in their claims.

If an illustration were needed, a case which recently occurred in Philadelphia will admirably answer. Two of the leading pathologists of the old school—one of them boasts that he can determine the actual condition of the internal organs as well as though they were laid open before him—were treating a gentleman for a painful affection in the region of the transverse colon. After weeks of drugging with Morphia and other infernal preparations there was no relief. They then declared that the liver was at fault, and that it would be necessary to cut down to that organ in order to rid it of—who knows what? The operation was performed and the liver was found all right. The gentleman lives to tell the tale, and to say that inflammation of the colon was present, and that he has had enough of scientific (?) medicine. Any Hahnemannian can bear testimony to the same effect. We have always declared that it is better never to affirm positively about the condition of any organ. We may believe that the symptoms present in any given case may point to some special organ or part; but we cannot be sure we are right unless it be demonstrated *post-mortem*—and that is a chance seldom offered to the genuine follower of Hahnemann. G. H. C.

---

PHYSIOLOGY.—The physiological factor, too, is of importance. We are in need of a physiology stripped of all hypothesis; giving only that which is known. With such a work we should be able to more fully appreciate the beauties of Hahnemannian Homœopathy.

"By means of pure observation and unprejudiced reflection, in connection with anatomy, natural philosophy, and chemistry, we have a considerable store of very probable conclusions regarding the operations and vital phenomena of the human body (*physiology*), because the phenomena in what is called a healthy body remain pretty constant, and hence can be observed frequently, and, for purposes of comparison, from all the different points of view afforded by the various branches of knowledge bearing upon them. But it is no less true, than striking and humbling, that this anthropological or physiological knowledge begins to prove of no use as soon as the system departs from its state of health. All explanations of *morbid* processes from what we know of healthy ones are deceptive, approaching more or less to what is untrue; at all events, positive proofs of the reality and truth of these transferred explanations are unattainable; they are from time to time refuted by the highest of all tribunals—experience. Just because an explanation answers for the healthy state of the frame, it will not answer for the diseased. We may admit it or not as we please, but it is too true that in the moment when we attempt to regard the state of the disease physiologically, there drops before our previous clear light of physiology a thick veil—a partition which prevents all vision. Our physiological skill is quite at fault when we have to explain the phenomena of morbid action. There is almost no part of it applicable! True, we can give a sort of far-fetched explanation by making a forced transference and application of the physiological systems to pathological phenomena; but it is only illusory and misleads into error.

"*Chemistry* should never attempt to offer an explanation of the abnormal performances of the functions in the diseased body, since it is so unsuccessful in explaining them in the healthy state. When it predicts what, according to its laws, must happen, then something quite different takes place; and if the vitality overmasters chemistry in the healthy body, how much more must it do so in the diseased, which is exposed to the influence of so many more unknown forces. And just as little should chemistry undertake to give a decision upon the suitableness or worthlessness of medicines, for it is altogether out of its sphere of vision to determine what is properly healing or hurtful, and it possesses no principle and no standard by which the healing efficacy of medicines in different diseases can be measured or judged.

"Thus has the healing artist forever stood alone—I might say forsaken—forsaken by all his renowned auxiliary sciences—forsaken by all his transcendental explanations and speculative systems. All these assistants were mute, when, for example, he stumbled upon an intermittent fever which would not yield to purgatives and cinchona bark.

"'What is to be done here? what is with *sure confidence* to be set about?' he inquires of these oracles. Profound silence. (And thus they remain silent up to the present hour, in most cases, these fine oracles)."

These words were written eighty-five years ago by a man who has since been termed "the sage of Coethen." Can they be successfully refuted to-day? G. H. C.

---

"QUININE drove a victim of influenza to suicide in Hartford, and many physicians are inclined to the belief that the use of this drug in excess is worse than the disease."—*N. Y. Commercial Advertiser*.

If the history of the old-school and mongrel treatment of this epidemic could be placed in the hands of intelligent people, we should like to know how many followers they would have. The same idea will apply to their treatment of all diseases. We wish that the people would read old-school journals. In many of them we frequently find cases in which the writers acknowledge that the treatment was undoubtedly the cause of death. They are honest in admitting the fact to those who read the journals, but their to-be-pitied patients are never made the wiser. G. H. C.

---

STRONG MEDICINE.—"By treatment the *ordinary* physician often understands nothing more than a powerful, violent attack upon the body with things that are to be found in the chemist's shop, with an alteration of the diet, *secundem artem*, to one of a very extraordinary, very meagre character. 'The patient must first be powerfully affected before I can do him any good; I wish I could but once get him regularly laid up in bed!' But then the transition from bed to the straw and the coffin is so very easy; infinitely easier than to health; he says nothing about that.

"The physician of the *stimulating* school is in the habit of prescribing in almost every case an exactly opposite diet (such is the custom of his sect): ham, strong meat soups, brandy, etc., often in cases where the very smell of meat makes the patient sick and he can bear nothing but cold water; but he is also by no means sparing in his use of violent remedies in enormous doses.

"The schools of both the former and the latter class authorize a revolutionary proceeding of this sort. 'No child's play with your doses,' say they; 'go boldly and energetically to work, giving them strong, as strong as possible!' And they are right, if treating means the same thing as knocking down." Hahnemann, 1805. G. H. C.

---

PROGRESSIVE HOMŒOPATHY.—

> "Under fair pretense of friendly ends,
> And well-placed words of glossy courtesy,
> Bated with reason not unplausible,
> Wind into easy-hearted men,
> And hug them into snares."

Mongrels are now masquerading under the name of "progressive homœopathists." With the name of "progressive Homœopathy" those who know little or nothing of Hahnemannian Homœopathy are being deluded. This title of "progressive Homœopathy" means only mongrelism and nothing more. Those who are familiar with the doing of this class need no warning; but those who are unfortunate enough to fall into their hands should be put on their guard, and made to know the genuine. Taking the fair name of Homœopathy, and using it for gain, while doing all that is possible to bring upon it discredit, "these dishonest men are living a lie." No Hahnemannian should hesitate to denounce them whenever and wherever they may be found.

The true physician has only the welfare of his patients at heart; the pretender thinks only of gain, regardless of the good of the patient. The "progressive homœopathist" claims that his use of Morphia for the relief of pain is a sign that he has broken away from the fossils, and that he is always doing what is possible for his patient. The Hahnemannian, on the other hand, conscientiously notes and studies all the signs and symptoms found in his patients, and then patiently goes to work to find the curative. The difference between the two is simply the distinction between the true and the false. The one is an honest man conscious of right. The other is anything but honest. No upright man professing a knowledge of the law of Homœopathy can do otherwise than adhere to that law under all circumstances in which the law is applicable. And it is applicable in all idiopathic and non-surgical affections. Even employed in surgical affections it is powerful for good. The use of drugs which are harmful in a crude form is no part of Homœopathy, and is to be decried. Except as an antidote, the Hahnemannian never uses such agents. He knows that he possesses remedies so prepared that they have no capacity for harm. With these weapons he is able to combat the most malignant, the most painful, and the most serious maladies that ever appear.

And he knows, furthermore, that he holds these agents for good for all time. He will not magnify one remedy above another for a little period, and then lay it aside for something new. He knows that the law which governs him is immutable, and that what is proper for a given train of symptoms to-day will be proper for similar symptoms to the end of time. A law of nature is unchangeable.

On the other hand, the drugger is without a guide, and his only thought is to make symptoms disappear by the use of crude drugs, and thus lead the patient and his friends to believe that he has done good. If he be the holder of any knowledge of Homœopathy he knows that his treatment has only done injury.

On this point we shall let the Sage of Coethen speak :

To Mongrels and Allopaths.—" Be not too anxious, I advise you, to insist on the dissection of the corpses of those you have done to death ! You would not do it did you know what you thereby revealed to him who knows the truth ! Besides, some rare congenital malformations, and perchance some results of the deceased's dissipation, what of an abnormal character do you encounter that is not chiefly the product of your injurious operations, of your medicinal ignorance and your therapeutic sins of omission and commission ? There is displayed *not what was present before your treatment*, as you would fain persuade the relatives, *but what was produced by your treatment—the incurability of the deceased was not before* but *after* your treatment. It avails you nothing, that you thereby gladly take the opportunity of making a display of your subtle anatomical terminological learning, neither can it be concealed from those who have any knowledge that this is no test of ability to cure. The result of such autopsies is not the enriching of pathological anatomy, *but the revelation of hideous therapeutic anatomy, to your disgrace*—in spite of all your plausible sophistries!" 1830.

